# War and the Anti-Tuberculosis Crusade: The Effect of Military Enlistment

**Published:** 1915-02-27

**Authors:** 


### THE WAR AND THE ANTI-TUBERCULOSIS CRUSADE.

### The Effect of Military Enlistment.

Remembering the intelligent prognostications, supported by incontrovertible arguments, which had been made concerning the effects which would follow upon the involvement of this country in war with a Continental Power, wonderment is still constantly expressed at the comparatively slight and superficial disturbance caused to our national life during six months of a colossal European war. The observer has to look below the surface to appreciate the national, and especially the economic and domestic, changes which have occurred and are occurring around him.

The war, whatever may have been the anticipations concerning it, broke upon the country at a time when favourable trade conditions and the trend of legislation had already permitted the development of many humanitarian and altruistic democratic schemes and the initiation of others. Questions of public health, in the widest sense of the term, were in the ascendant, and enormous sums of money were being diverted in the interests of what might have been called, before the term became opprobrious, national " culture."

The most important of recent developments was the passing of the Insurance Act, 1911, with its revolutionary provisions concerning medical treatment, including the treatment of tuberculosis. Upon the financial basis of the Act depends the fact that, although its machinery was yet in the making at the time when the war arose, so slight an interference has been caused to its evolution.

### Temptation to Mark Time.

What has occurred is less a stoppage of activities under the Act than (to use a military phrase) a tendency, wherever possible, to mark time until the opportunity for progress is more favourable. This tendency reveals itself in such matters as the suspension of grants in aid of nursing schemes, for which, indeed, it would be now difficult to find nurses. Some Insurance Committees, too, having in mind reduction of receipts due to the diversion of large numbers of men from ordinary to military occupations, are suspending payments to medical practitioners. It may be remarked, and the fact should not be forgotten, that these medical practitioners are in many cases gratuitously attending the dependants of men on military service.

As to the work of the anti-tuberculosis campaign, arguments in favour of curtailing expenditure, postponing building of sanatoria, etc., whilst they have appealed to the cautious, have also been readily seized upon by those who are timorous of, or who are actually opposed to, actively administering the provisions of the Act. The medical staff of the tuberculosis dispensaries and of institutions for the tuberculous has been reduced by withdrawals incident to the present shortage of medical men and to the demands of military service, etc. Whilst such withdrawals have not been sufficiently numerous to cripple the service, great difficulty is being experienced in suitably filling vacancies (many of which are being advertised) for tuberculosis medical officers and assistants. Postponement in making new appointments is frequent.

The work of the crusade and of those engaged in it has been directly affected by enlistment for military service in a far greater degree than was anticipated or could have been foreseen. Large numbers of men who were under treatment or supervision, who were known to be or were suspected of being tuberculous, or who had been under treatment in sanatoria, have enlisted. Although in a few instances this acceptance of the hardships and exposure of active service has been unwise, has been undertaken in spite of medical advice, and might even constitute a source of danger to others, the results have been upon the whole satisfactory and encouraging, and the proportion in which these patients and suspects have broken down is relatively and indeed surprisingly small.

### Tuberculosis after the War.

There naturally arises the question whether, amongst those who have served in the Navy or Army during this unprecedented war, a high percentage of cases of tuberculous disease will arise after return to other conditions of life. We do not believe that active service will prove to cause wide predisposition to tuberculous invasion. There was a period during which the conditions of military, and still more of naval, service were highly conducive to tuberculosis. The evils of cold and wet, of alternations of exposure with the use of overcrowded and ill-ventilated sleeping-places, of defective teeth, of insufficient and unsuitable food, of the use as hospitals of buildings quite unsuitable and unprepared—all have been in this war in a remarkable degree eliminated or reduced.

Dr. Murray Leslie, in a lecture recently delivered at the Institute of Hygiene, has called attention to the physical advantages gained by recruits now under training and the general improvements shown by them in stamina, endurance, capacity for sustained effort, and power of resistance to disease. Granted that height, weight, and chest measurement (that is to say, lung capacity) are duly proportioned, the hardships of training at all events can be borne without undue risk to health, and often ultimately prove even beneficial. Amazement is expressed at the extraordinary development which has taken place in flat chested, somewhat weedy-looking young men as a result of a few months' military training; some " improved out of all knowledge." Very definite hygienic advantages (precisely similar in character to those attributed in this country to athletic training) are claimed in Japan, France, and Russia to result from universal military training. Much importance has been claimed for work done in Germany with a view to the prevention of tuberculosis. The following quotation from " Eye-

Witness" (the *Times*, Saturday, February 13) is, therefore, not irrelevant: "It has already been mentioned that some of the prisoners captured by us lately have been of comparatively poor physique. In this connection it is interesting to note that during the last few days a dead German was found having two medical certificates in his pocket stating that he was suffering from consumption. They are both signed by a doctor, and are accompanied by an application from his father that his son should not be sent on active service, as he was suffering from lung trouble."

